# Additional predictive value of optic nerve sheath diameter for neurological prognosis after cardiac arrest: a prospective cohort study

**DOI:** 10.1186/s13089-023-00344-3

**Published:** 2023-12-08

**Authors:** Marlous M. L. H. Verhulst, Iris M. Visser, Hanneke M. Keijzer, Nicole L. M. de Kruijf, Erwin J. G. Peters, Thom Wilbers, Roel V. Peelen, Jeannette Hofmeijer, Michiel J. Blans

**Affiliations:** 1 Department of Neurology, Rijnstate Hospital, 6800 TA Arnhem, The Netherlands; 2 Department of Clinical Neurophysiology, Faculty of Science and Technology, University of Twente, 7522 NB Enschede, The Netherlands; 3 Technical Medicine, University of Twente, 7522NB Enschede, The Netherlands; 4 Department of Intensive Care Medicine, Rijnstate Hospital, 6800 TA Arnhem, The Netherlands

**Keywords:** Optic nerve sheath diameter, Cardiac arrest, Prognosis, Ultrasound, Neurological outcome, Intracranial pressure

## Abstract

**Background:**

The goal is to estimate the additional value of ultrasonographic optic nerve sheath diameter (ONSD) measurement on days 1–3, on top of electroencephalography (EEG), pupillary light reflexes (PLR), and somatosensory evoked potentials (SSEP), for neurological outcome prediction of comatose cardiac arrest patients. We performed a prospective longitudinal cohort study in adult comatose patients after cardiac arrest. ONSD was measured on days 1–3 using ultrasound. Continuous EEG, PLR, and SSEP were acquired as standard care. Poor outcome was defined as cerebral performance categories 3–5 at 3–6 months. Logistic regression models were created for outcome prediction based on the established predictors with and without ONSD. Additional predictive value was assessed by increase in sensitivity for poor (at 100% specificity) and good outcome (at 90% specificity).

**Results:**

We included 100 patients, 54 with poor outcome. Mean ONSD did not differ significantly between patients with good and poor outcome. Sensitivity for predicting poor outcome increased by adding ONSD to EEG and SSEP from 25% to 41% in all patients and from 27% to 50% after exclusion of patients with non-neurological death.

**Conclusions:**

ONSD on days 1–3 after cardiac arrest holds potential to add to neurological outcome prediction.

*Trial* *registration*: clinicaltrials.gov, NCT04084054. Registered 10 September 2019, https://www.clinicaltrials.gov/study/NCT04084054.

**Supplementary Information:**

The online version contains supplementary material available at 10.1186/s13089-023-00344-3.

## Background

Neurological outcome prediction after cardiac arrest is challenging. International guidelines recommend using a combination of absent pupillary and corneal reflexes, bilaterally absent somatosensory evoked potentials (SSEP), highly malignant electroencephalogram (EEG) patterns, neuron-specific enolase > 60 µg/L, status myoclonus, and diffuse and extensive anoxic injury on brain CT or MRI [1]. Based on these, reliable prediction of a poor outcome is possible in 32–47% of all patients [2–4]. This implies ongoing uncertainty in 53–68%, indicating a high demand for additional bedside measurements that contribute to neurological outcome prediction of comatose patients after cardiac arrest.

Raised intracranial pressure (ICP), resulting from brain oedema, can contribute to poor neurological outcome after cardiac arrest [5]. However, direct ICP measurements are undesirable because of invasiveness and possible risks. Various studies have shown that a large optic nerve sheath diameter (ONSD) is associated with raised ICP in diverse patient groups [6–10], including patients with hypoxic–ischaemic brain damage after cardiac arrest [11]. This association can be explained by the extension of the subarachnoid space along the optic nerve within the sheath, resulting in expansion of the optic nerve sheath in case of increased ICP [9, 12]. The ONSD can be measured using bedside ultrasound, which is harmless, inexpensive, and quick (5–10 min). Previous research showed that a large ONSD is associated with poor neurological outcome after cardiac arrest [13–19], but the additional predictive value and optimal timing of ONSD, on top of currently recommended predictors, is still unclear.

The aim of the current study was to assess the value of ultrasonographic ONSD measurements on days 1–3, in addition to continuous EEG measurements, pupillary light reflexes (PLR), and SSEP, for prediction of neurological outcome of comatose patients after cardiac arrest.

## Methods

### Study design

We performed a prospective cohort study at Rijnstate hospital, The Netherlands. Consecutive comatose patients after cardiac arrest were included for daily ONSD measurement, in addition to standard care. Patients were included between December 2019 and October 2021. The medical ethics committee Arnhem–Nijmegen approved the study protocol and waived the need for informed consent prior to study inclusion (2019–5586). In case of patient survival up to 72 h, deferred consent was obtained from the patient and/or relatives. The study is registered (ClinicalTrials.gov identifier: NCT04084054).

### Study population

Consecutive comatose patients after cardiac arrest (in-hospital and out-of-hospital) were included within 24 h after cardiac arrest. Inclusion criteria were: Glasgow Coma Scale (GCS) ≤ 8 at admission, age ≥ 18 years, and admission to the intensive care unit (ICU). Exclusion criteria were pregnancy, traumatic brain injury, relevant eye surgery in medical history, pre-existing dependency in daily living (cerebral performance category (CPC) 3–4), or any known progressive brain illness, such as a brain tumour or neurodegenerative disease.

### Standard of care

Patients were monitored and treated according to local protocols that were in line with international guidelines for comatose patients after cardiac arrest [1]. Targeted temperature management at 36 °C was induced as soon as possible after arrival at the ICU and maintained for 24 h. After 24 h, passive rewarming was controlled and normothermia was actively maintained. Patients generally received a combination of propofol, midazolam, and morphine for sedation and analgesia.

### Decisions on withdrawal of treatment

Withdrawal of life sustaining treatment (WLST) was considered at ≥ 72 h after cardiac arrest, during normothermia, and off sedation. Decisions on WLST were based on European guidelines [1, 20] at the discretion of the treating physicians. The ONSD was never included in decisions on WLST and treating physicians were blinded to ONSD measurements.

### Neurological outcome

Neurological outcome was assessed at 3–6 months after cardiac arrest by a standardized telephone interview by one of two researchers (MV, HK), blinded to ONSD measurements, according to the cerebral performance categories (CPC). Neurological outcome was dichotomized as “good” (CPC 1–2: no to moderate disability) or “poor” (CPC 3–5: severe disability, vegetative state, or death).

### Study endpoints

The primary study endpoint is increase in sensitivity for predicting poor and good outcome after adding ONSD measurements to established parameters. Secondary endpoints include feasibility, inter- and intra-observer reliability, and differences in ONSD between patients with good and poor outcome.

### Data acquisition and analysis

#### EEG

Continuous EEG recordings were started in all patients as soon as possible after arrival at the ICU, always within 24 h after cardiac arrest, and continued for at least 3 days or until a patient’s decease or awakening, as part of standard care. Twenty-one electrodes were placed on the scalp according to the international 10–20 system. EEG recordings were performed using a Nihon Kohden system (VCM Medical, The Netherlands) from the study start to March 2021 and a BrainRT system (OSG, Belgium) from April 2021 onwards. Two reviewers (MV, HK) independently classified anonymized EEG epochs at 6, 12, 24, 36, 48, and 72 h after cardiac arrest blinded to the timing of the epoch, a patient’s clinical status, medication, and outcome. In case of disagreement, consensus was obtained by the consultation of a third reviewer (JH). EEG patterns were classified as suppressed with or without superimposed synchronous activity, continuous, or other patterns [4].

#### SSEP

SSEP recordings were performed off-sedation using a Nicolet EDX system (Natus Medical Inc., USA) as part of standard care at the treating physician’s request, generally between 48 and 72 h in patients who remained comatose after restoration of normothermia. Bilaterally absence of N20 responses was considered predictive of poor neurological outcome.

#### Pupillary light reflexes (PLR)

PLR were tested daily by treating physicians and categorized as present or bilaterally absent. Bilaterally absent PLR > 72 h after cardiac arrest was considered predictive of poor neurological outcome.

#### ONSD

**Fig. 1:**
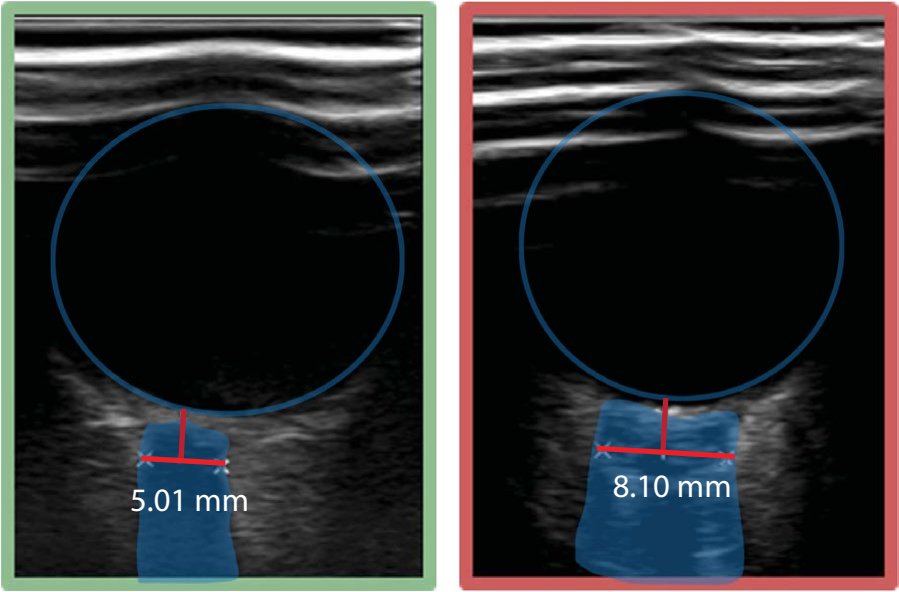
Examples of ultrasound images of the ONSD for a patient with good neurological outcome (left, ONSD = 5.01 mm) and a patient with poor neurological outcome (right, ONSD = 8.10 mm). The eyeball and optic nerve including its sheath are delineated in blue. The red horizontal line indicates the ONSD

ONSD was measured daily by trained personnel in the first 3 days after cardiac arrest, or until decease or awakening. Three consecutive measurements per eye were performed each day using an Affiniti 70C ultrasound system (Philips, The Netherlands). A linear probe with a frequency range of 3–12 MHz was used. Sterile ultrasound gel was placed on the probe and a sterile probe cover was placed over it, preventing ultrasound gel from touching the eye. The probe was placed transversally on the superior lateral part of the upper eyelid, angled caudally and medially with the head of the patient 30° elevated. No pressure was put on the eye. The field was reduced to a depth of 4 cm. The ONSD was measured 3 mm behind the retina [21] at the transition from the hyperechoic retrobulbar fat to the hypoechoic line, in the presence of hyperechoic striped bands, or at the transition from the hyperechoic retrobulbar fat to the hypoechoic region of the optic nerve, in absence of striped bands. These marker placements both correspond to the outer edges of the dura mater [22] (Fig. 1). The mean of three binocular ONSD measurements per day was used for further analysis.

### Statistical analyses

Data are presented as mean ± standard deviation for continuous normally distributed data or median with interquartile range [IQR] for non-normally distributed data. To compare patients with good and poor outcome on a group level, we used Chi-squared tests for ordinal, and unpaired *t* tests or Mann–Whitney U tests for continuous variables.

Inter-observer reliability was assessed based on the ultrasound images of 10% of the included patients (*n* = 10), who were selected at random. One reviewer (MV) re-measured the ONSD of these patients offline, blinded for the original measurement. Intra-observer reliability was assessed based on three consecutive measurements per eye per day. Inter-observer and intra-observer reliability were calculated using the intraclass correlation coefficient (ICC) based on a two-way mixed-effects model with absolute agreement [23].

To test the additional predictive value of ONSD measurements on top of established parameters, we created a logistic regression model and a mixed-effects logistic regression model. The logistic regression model included two categorical variables (EEG classified as suppressed after 24 h, continuous within 12 h, or other; SSEP classified as not absent (not tested or present) or absent). PLR was excluded from the analysis because of the low frequency (*n* = 1) of bilaterally absent PLR in our cohort. ONSD measurements were normalized to *z*-scores. A mixed-effects logistic regression model with random intercept was trained (70% of data) and validated (30% of data). Fixed effects were the EEG, SSEP, ONSD, and time (days 1, 2 or 3). Study ID was used as a random effect. Predictive values of the models were evaluated using receiver operating characteristics (ROC): area under the curve (AUC), sensitivity to predict poor outcome at 100% specificity, sensitivity to predict good outcome at 90% specificity, and positive and negative likelihood ratios for predicting poor and good outcome. Additional predictive value of ONSD measurements was assessed by an increase in sensitivity for predicting poor and good outcome and a decrease in the Akaike information criterion (AIC). We checked for multicollinearity between our predictors using the variance inflation factor (VIF). Multicollinearity was assumed if VIF ≥ 5. Additional predictive value of ONSD measurements was assessed in the full data set and a subset (after exclusion of patients with a non-neurological cause of death). Sample size calculations were based on 10 patients per outcome group for every predictor added to the model. Adding 5 predictors (ONSD day 1, day 2, day 3, EEG, and SSEP), indicated the need of 100 patients (with an expected distribution of good vs. poor outcome of 50/50).

*P* values < 0.05 were assumed statistically significant. All statistical analyses were performed using R version 4.0.0.

## Results

**Fig. 2:**
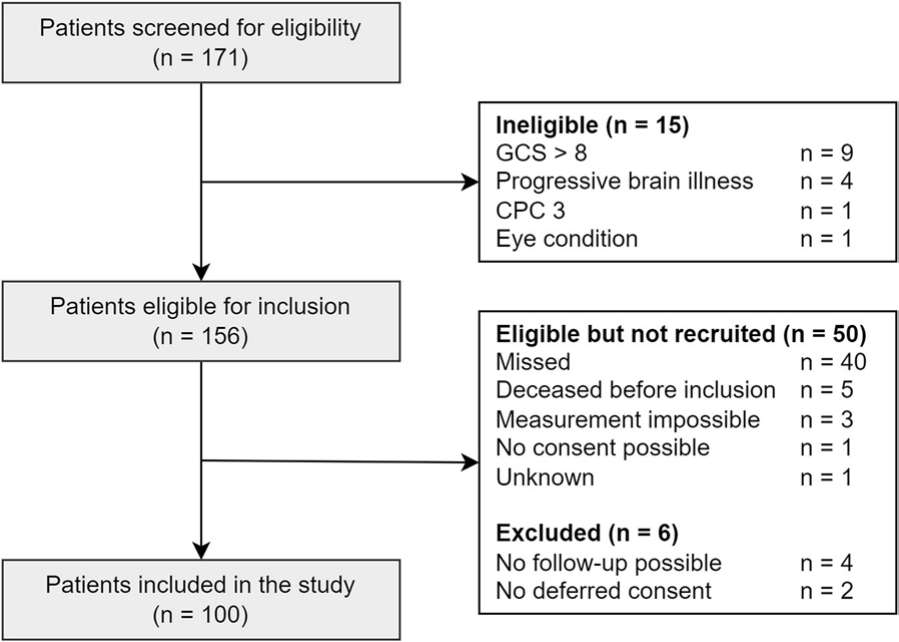
STROBE (STrengthening the Reporting of Observational studies in Epidemiology) flow diagram outlining the selection of adult patients admitted to the ICU after a cardiac arrest. *CPC* cerebral performance category, *GCS* Glasgow Coma Scale

We screened 171 patients on the ICU after a cardiac arrest and included 100 patients (Fig. 2), of whom 54 (54%) had a poor neurological outcome. Baseline characteristics are presented in Table 1. Patients with poor outcome were older, had a non-shockable first rhythm more often, and had longer times to return of spontaneous circulation (ROSC) than patients with good outcome. The SSEP N20 response was bilaterally absent in six patients with poor outcome and never in patients with a good outcome. A continuous EEG within 12 h (suggestive of a good outcome) was seen in ten patients with good outcome and two patients with poor outcome [4] These two patients died of secondary hemodynamic or neurological decline. A suppressed EEG pattern with or without superimposed synchronous activity later than 24 h after cardiac arrest (suggestive of a poor outcome) was observed in three patients with poor outcome and never in patients with a good outcome [1, 4].

**Table 1:** Baseline characteristics of study population

	Good outcome (*n* = 46)	Poor outcome (*n* = 54)	*P* value
Age (years)	58.6 ± 10.0	69.7 ± 10.3	** < 0.01**
Male	34 (74%)	38 (70%)	0.87
OHCA	45 (98%)	51 (94%)	0.73
Shockable first rhythm	45 (98%)	37 (69%)	** < 0.01**
Time to ROSC (minutes)	15 (10–20)	19 (15–30)	** < 0.01**
Absent PLR ≥ 72 h	0 (0%)	1 (2%)	1.00
SSEP			** < 0.01**
Bilaterally absent N20	0 (0%)	7 (13%)	
Present N20	2 (4%)	20 (37%)	
Not tested	44 (96%)	27 (50%)	
EEG			** < 0.01**
Suppressed > 24 h	0 (0%)	5 (9%)	
Continuous < 12 h	13 (28%)	3 (6%)	
Inconclusive	33 (72%)	46 (85%)	

Data are presented as *n* (%) for dichotomous variables, mean ± standard deviation for normally distributed continuous variables, and median [interquartile range] otherwise. *EEG* electroencephalogram, *OHCA* out-of-hospital cardiac arrest, *PLR* pupillary light reflexes, *ROSC* return of spontaneous circulation, *SSEP* somatosensory evoked potentials

### Feasibility

ONSD measurements were performed in 37 (80%), 28 (61%), and 16 (35%) patients in the good outcome group, and in 39 (72%), 33 (61%), and 25 (46%) patients in the poor outcome group on days 1, 2, and 3, respectively. All patients underwent ONSD measurements on at least one of these days. Most important reasons for missing measurements were regaining consciousness in the good outcome group, and decease in the poor outcome group (Table 2).

**Table 2:** Reasons for missing measurements in both outcome groups for days 1, 2, and 3

	Good outcome (*n* = 46)			Poor outcome (*n* = 54)		
	Day 1	Day 2	Day 3	Day 1	Day 2	Day 3
Logistic reasons	8	3	2	14	7	3
Conscious or off sedation	0	14	27	0	4	4
Deceased or treatment withdrawal	0	0	0	0	10	20
Unsuccessful measurement	0	0	0	1	0	0
Transferred to other hospital	0	1	1	0	0	1
Instability	1	0	0	0	0	1
Total	9 (20%)	18 (39%)	30 (65%)	15 (28%)	21 (39%)	29 (54%)

### Inter- and intra-observer reliability

Inter-observer reliability was assessed in 118 images of 10 randomly selected patients. The mean difference between offline and real-time measurements of the ONSD was 0.006 mm with upper and lower bounds 0.52 and − 0.49 mm, respectively. The intraclass correlation coefficient for offline and real-time measurements (inter-observer reliability) was 0.872 (95%CI 0.816–0.911). Intra-observer reliability for three consecutive measurements per eye was 0.919 (95% CI 0.903–0.932).

### ONSD in good and poor outcome

**Fig. 3:**
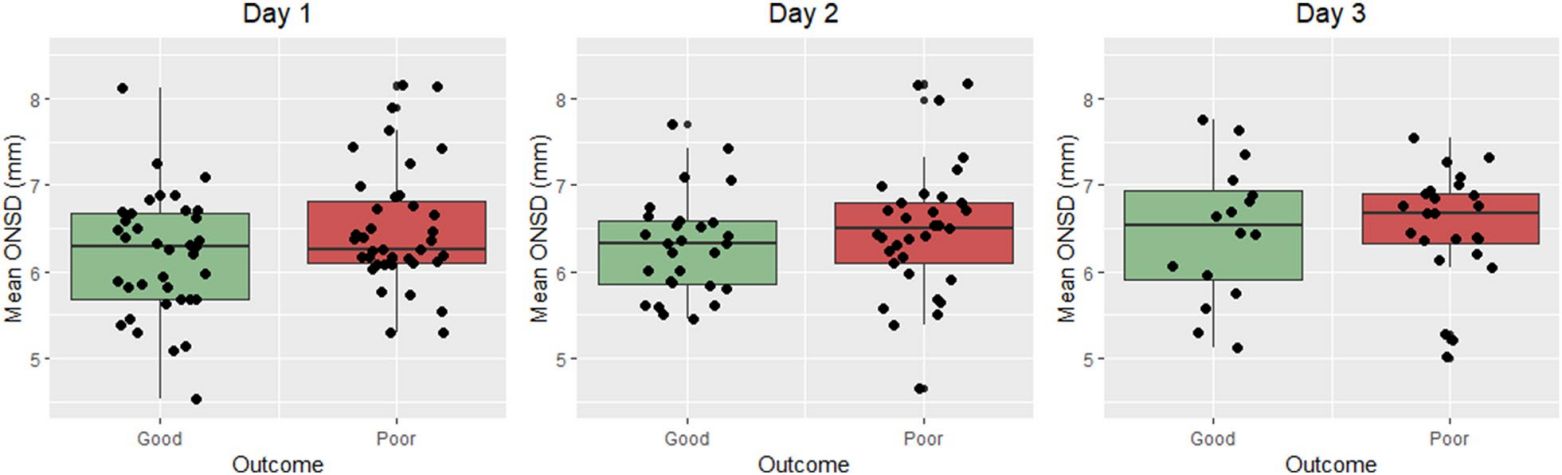
Boxplots showing the mean binocular measured ONSD on days 1, 2 and 3 for patients with good (green) and poor (red) outcome. Dots represent mean binocular ONSD for individual patients. Boxplots show no statistically significant differences between patients with good and poor outcome on days 1–3

Mean binocular ONSD values are presented in Fig. 3 and Additional file 1: Table S1. No statistically significant differences in mean ONSD were found between patients with good and poor outcome on any day after cardiac arrest.

### Predictive values

#### Full data set

**Fig. 4:**
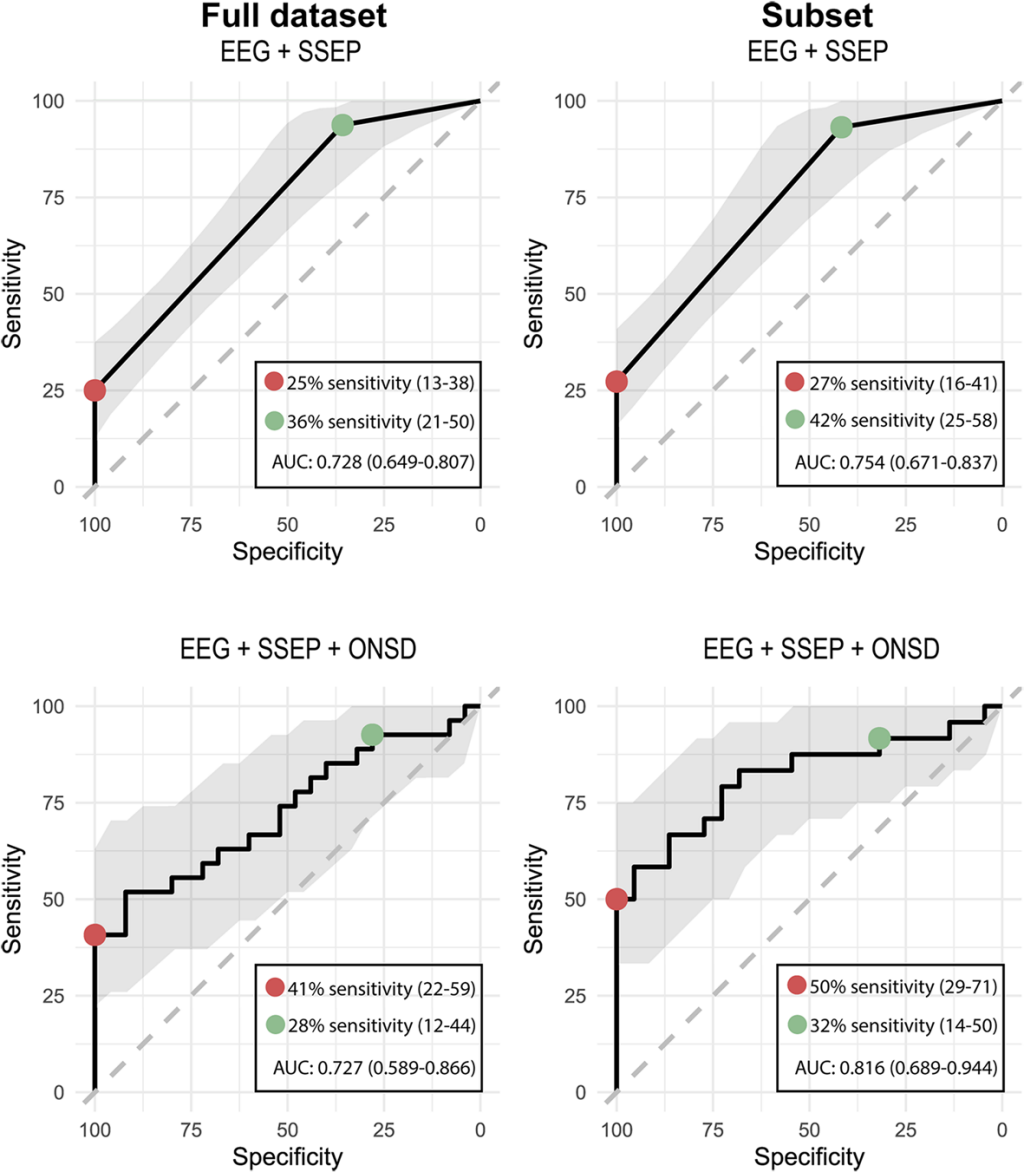
ROC curves for logistic regression models based on EEG and SSEP (upper row), and mixed models after adding ONSD on days 1, 2, and 3 (lower row). Left column shows ROC curves for the full data set, right column for the subset (after exclusion of patients with non-neurological cause of death). The red and green circles indicate the thresholds for predicting poor and good neurological outcome, respectively. *AUC* area under the curve, *EEG* electroencephalogram, *ONSD* optic nerve sheath diameter, *SSEP* somatosensory evoked potentials

Additional predictive values of ONSD measurements for outcome were evaluated using ROC analyses. AUC for the prediction model based on EEG and SSEP was 0.728 (95% CI 0.649–0.807). The AUC did not change after adding the ONSD measurements (0.727 (95% CI 0.589–0.866)). Otherwise, sensitivity for prediction of poor outcome increased from 25% (95% CI 13–38%) to 41% (95% CI 22–59%) at 100% specificity after adding the ONSD measurements. Sensitivity for prediction of good outcome decreased from 36% (95% CI 21–50%) to 28% (95% CI 12–44%) at 90% specificity. The AIC was 253.4 for the prediction model on EEG and SSEP, and decreased to 147.4 after adding the ONSD measurements. ROC curves including sensitivities and specificities for the models based on EEG and SSEP, and based on EEG, SSEP, and ONSD are shown in Fig. 4 (left column). Likelihood ratios for poor outcome indicated a decrease in probability of a poor outcome in case of the absence of a poor prediction after adding the ONSD measurements (LR - 0.75 vs. 0.59). In addition, likelihood ratios for good outcome indicated an decrease in probability of a good outcome in case of a good prediction (LR + 6.00 vs. 4.00), and an increase in probability of a good outcome in case of the absence of a good prediction after adding the ONSD measurements (LR - 0.68 vs. 0.77). A complete overview of AUC, sensitivity for prediction of poor outcome at 100% specificity, sensitivity for prediction of good outcome at 90% specificity, and likelihood ratios of the various models is shown in Additional file 1: Tables S2 and S3. A summary of the logistic regression model and mixed effects model are shown in Additional file 1: Tables S4 and S5, respectively. The VIF between predictors was never above 5, indicating no to moderate correlation.

#### Subset excluding non-neurological cause of deaths

After exclusion of patients with a non-neurological cause of death (*n* = 12), AUC for the prediction model based on EEG and SSEP was 0.754 (95% CI 0.671–0.837). The AUC increased to 0.816 (95% CI 0.689–0.944) after adding the ONSD measurements to the model. Furthermore, the sensitivity for predicting poor outcome increased from 27% (95% CI 16–41%) to 50% (95% CI 29–71%) at 100% specificity after adding the ONSD measurements. Sensitivity for prediction of good outcome decreased from 42% (95% CI 25–58%) to 32% (95% CI 14–50%) at 90% specificity. The AIC was 191.9 for the prediction model on EEG and SSEP, and decreased to 116.2 after adding the ONSD measurements. ROC curves including sensitivities and specificities for the model based on EEG and SSEP, and the models with mean binocular ONSD on days 1–3 are shown in Fig. 4 (right column). Likelihood ratios for poor outcome indicated a decrease in probability of a poor outcome in case of the absence of a poor prediction after adding the ONSD measurements (LR− 0.73 vs. 0.50). In addition, likelihood ratios for good outcome indicated an decrease in probability of a good outcome in case of a good prediction (LR + 6.00 vs. 4.00), and an increase in probability of a good outcome in case of the absence of a good prediction after adding the ONSD measurements (LR - 0.62 vs. 0.74). AUC and sensitivities for prediction of poor and good outcome were higher in the subset analyses compared to the analyses based on the full data set. A summary of the logistic regression model and mixed effects model are shown in Additional file 1: Tables S6 and S7, respectively.

## Discussion

In our cohort of comatose patients after cardiac arrest, adding ONSD measurements to SSEP and EEG increased the sensitivity for reliable prediction of poor outcome from 25% to 41%. After exclusion of patients with a non-neurological cause of death, sensitivity for reliable prediction of poor outcome increased from 27% to 50%. Prediction of good neurological outcome did not improve by adding ONSD measurements.

To the best of our knowledge, this study is the first to investigate the additional predictive value of repeated ONSD measurements for neurological outcome after cardiac arrest, on top of continuous EEG and SSEP. Previous studies mainly focused on the predictive value of ONSD measurements alone or on top of parameters not included in current guidelines [14, 16, 17, 24]. These studies reported mean ONSD between 3.78 and 6.7 mm for patients with good outcome and between 4.88 and 7.3 mm for patients with poor outcome [14–18, 24–26]. ONSD values below 5.4 mm were predictive of good outcome [14], and ONSD values above 5.11 to 7.0 were predictive of poor outcome [16–18, 26]. This is mostly in line with our results, although our ONSD values were rather large compared to some studies. This might be explained by the differences in marker placement on the ultrasound images. Agreement on the appearance of the pia mater, the subarachnoid space, and the dura mater is lacking [22]. We placed our markers at the transition from the hyperechoic retrobulbar fat to the hypoechoic line or at the transition from the hyperechoic retrobulbar fat to the hypoechoic region of the optic nerve, most likely corresponding to the outer edges of the dura mater. The diameters we found are in line with other studies using these edges for marker placement. We found no significant differences in ONSD between patients with good and poor neurological outcome. This is in line with some [24–26] but in contrast with other studies [13–18]. The timing of measurement was a non-significant predictor of outcome in the mixed effects model, indicating that the timing of measurement (days 1, 2, or 3) did not influence the prediction of neurological outcome in our cohort.

We found a large variance in ONSD measurements, which might be partly caused by human variability. This problem could be overcome by looking at changes of ONSD relative to pre-cardiac arrest ONSD [27]. Nonetheless, pre-cardiac arrest ONSD is usually not available. Another solution might be to use the ONSD relative to the eyeball diameter. However, previous research showed that absolute ONSD and ONSD/eyeball diameter ratio had comparable performance in predicting outcome after cardiac arrest [28]. Using colour-Doppler Ultrasound instead of B-mode Ultrasound might yield lower variability and lower mean ONSD values [29]. However, the comparison between these methods has not been made in our population.

We showed that ONSD measurements hold potential to add to poor neurological outcome prediction on top of EEG and SSEP. Likelihood ratios for poor outcome prediction indicated a large increase in probability of a poor outcome in case of a poor prediction, with similar likelihood ratios for the models with and without ONSD. The probability of a poor outcome in case of the absence of a poor prediction decreased after adding the ONSD measurements. Although the increase in sensitivity and decrease in negative likelihood for predicting poor outcome is small, this can be clinically relevant, because measurements are non-invasive, cheap, and fast. Furthermore, the AIC decreased considerably when adding the ONSD measurements to the model, indicating an improvement of the model for this data set. The possible additional value of ONSD measurements can be explained from a pathophysiological perspective: EEG and SSEP represent synaptic functioning and ONSD is an indirect measure of intracranial pressure. Methods to evaluate brain oedema (such as CT or MRI) require patient transportation to the radiology department, while ultrasonographic ONSD measurements can easily be performed at the bedside with negligible harm or risk for the patient. Therefore, clinical implementation of ONSD measurements is relatively easy, cheap, and time efficient compared with other imaging methods. A small increase in predictive value might, therefore, be already of clinical value.

Imaging studies have revealed that brain oedema typically manifests at 3–5 days after cardiac arrest [30, 31]. Still, we found the largest differences in ONSD between good and poor outcome patients and the highest predictive values on day 1. An explanation might be that patients without elevated ICP had likely regained consciousness after day 1 and many patients with severe brain damage deceased within 48 h in our cohort. This may explain that the spread in severity of brain damage was larger on day 1 compared to days 2 and 3, and that our population was smaller on days 2 and 3 (48 and 35 patients, respectively) compared to day 1 (60 patients). Conceptually, previous research showed that 70% of patients with poor neurological outcome showed a peak ICP > 15 mmHg within the first 24 h after cardiac arrest [5]. We speculate that other mechanisms than imageable oedema may contribute to increased ICP or ONSD after cardiac arrest.

Ultrasonic measurements are subjective and sensitive to inter- and intra-observer differences and should be performed by well-trained personnel. Still, the learning curve for ONSD measurements is steep and measurements can easily be learned and performed by ICU personnel [32]. The inter- and intra-observer reliability in our study were high (0.872 and 0.919, respectively). The mean absolute difference between real-time and offline measurements was 0.29 mm. However, we only evaluated inter-rater reliability based on online and offline measurements. We were not able to have two independent raters perform measurements on the same patient. A great part of the variance is likely introduced in the dynamic process of ultrasound imaging. The inter-rater reliability is, therefore, probably overestimated in our research and should be interpreted with care.

A strength of this research is the prospective design and inclusion of a broad selection of consecutive patients. We had few exclusion criteria, resulting in a patient population that likely represents the majority of comatose cardiac arrest patients. Furthermore, we used a training and test set to internally validate the results we found.

Our study also has some limitations. First, the time range of the measurements for each day was large. The exact moment of measurement (e.g., 3 or 23 h after cardiac arrest) might have influenced our measurements because of the strong time dependency of the pathophysiological processes going on in the brain during the first days after cardiac arrest. Second, measurements were performed by multiple sonographers. Despite careful training, we cannot exclude small differences in assessment between observers. Third, mean arterial pressure, partial arterial CO_2_ pressure, end-tidal CO_2_ and positive end-expiratory pressure levels might influence intracranial pressure and, therefore, ONSD at time of the measurements [33–35]. We did not take these clinical parameters into account. Fourth, we did not incorporate all predictors described in the European guidelines [1], since NSE measurements and brain imaging are not part of standard care for patients after cardiac arrest in our hospital. Fifth, part of the measurements were performed by the treating physicians for logistics reasons. ONSD measurements were never taken into account in decisions on patient treatment and we found a good inter-rater reliability. Therefore, we do not expect that this influenced our results. Finally, this is a single centre study. Clinical applicability warrants multicentre external validation of the results.

## Conclusions

In conclusion, ONSD measurement on days 1–3 after cardiac arrest provides a non-invasive bedside method that holds potential to add to poor neurological outcome prediction in addition to EEG and SSEP.

### Supplementary Information

**Additional file 1: Table S1.** Mean binocular ONSD measurements on days 1–3 for patients with good and poor neurological outcome. **Table S2.** Predictive values of EEG + SSEP and ONSD measurements based on logistic regression model (EEG + SSEP) and mixed model with random intercept (EEG + SSEP + ONSD) for the full data set and subset after exclusion of patients with non-neurological cause of death. **Table S3.** Likelihood ratios of EEG + SSEP and ONSD measurements based on logistic regression model (EEG + SSEP) and mixed model with random intercept (EEG + SSEP + ONSD) for the full data set and subset after exclusion of patients with non-neurological cause of death. **Table S4.** Results of logistic regression model for prediction of neurological outcome based on EEG and SSEP for the full data set. **Table S5.** Results of mixed effects model for prediction of neurological outcome based on EEG, SSEP, and ONSD measurements for the full data set. **Table S6.** Results of logistic regression model for prediction of neurological outcome based on EEG and SSEP for the subset (after exclusion of patients with a non-neurological cause of death). **Table S7.** Results of mixed effects model for prediction of neurological outcome based on EEG, SSEP, and ONSD measurements for the subset (after exclusion of patients with a non-neurological cause of death).

## Data Availability

The data sets used and/or analysed during the current study are available from the corresponding author on reasonable request.

## Abbreviations

AIC: Akaike information criterion
AUC: Area under the curve
CPC: Cerebral performance category
EEG: Electroencephalogram
GCS: Glasgow Coma Scale
ICC: Intraclass correlation coefficient
ICP: Intracranial pressure
ICU: Intensive care unit
ONSD: Optic nerve sheath diameter
PLR: Pupillary light reflexes
ROC: Receiver operating characteristic
ROSC: Return of spontaneous circulation
SSEP: Somatosensory evoked potential
VIF: Variance inflation factor
WLST: Withdrawal of life-sustaining treatment
