# Supplementary material for: Additional predictive value of optic nerve sheath diameter for neurological prognosis after cardiac arrest: a prospective cohort study
Source: Ultrasound J. 2023 Dec 8;15:46. doi: 10.1186/s13089-023-00344-3 (PMC10709263; doi:10.1186/s13089-023-00344-3)
Supplement: Supplementary file 1 — Additional file 1: Table S1. Mean binocular ONSD measurements on days 1–3 for patients with good and poor neurological outcome. Table S2. Predictive values of EEG + SSEP and ONSD measurements based on logistic regression model (EEG + SSEP) and mixed model with random intercept (EEG + SSEP + ONSD) for the full data set and subset after exclusion of patients with non-neurological cause of death. Table S3. Likelihood ratios of EEG + SSEP and ONSD measurements based on logistic regression model (EEG + SSEP) and mixed model with random intercept (EEG + SSEP + ONSD) for the full data set and subset after exclusion of patients with non-neurological cause of death. Table S4. Results of logistic regression model for prediction of neurological outcome based on EEG and SSEP for the full data set. Table S5. Results of mixed effects model for prediction of neurological outcome based on EEG, SSEP, and ONSD measurements for the full data set. Table S6. Results of logistic regression model for prediction of neurological outcome based on EEG and SSEP for the subset (after exclusion of patients with a non-neurological cause of death). Table S7. Results of mixed effects model for prediction of neurological outcome based on EEG, SSEP, and ONSD measurements for the subset (after exclusion of patients with a non-neurological cause of death). [file 13089_2023_344_MOESM1_ESM.docx]

**Additional materials**

Additional predictive value of optic nerve sheath diameter for neurological prognosis after cardiac arrest

**Table S1. Mean binocular ONSD measurements on day 1 to 3 for patients with good and poor neurological outcome.**

|  | Good outcome | Poor outcome | P-value |
| --- | --- | --- | --- |
| Day 1 | 6.29 [5.68-6.68] | 6.26 [6.11-6.82] | 0.17 |
| Day 2 | 6.32 [5.86-6.58] | 6.50 [6.09-6.80] | 0.21 |
| Day 3 | 6.54 [5.90-6.93] | 6.67 [6.33-6.91] | 0.84 |

*Data are presented as median [IQR].*

**Table S2. Predictive values of EEG + SSEP and ONSD measurements based on logistic regression model (EEG + SSEP) and mixed model with random intercept (EEG + SSEP + ONSD) for the full dataset and subset after exclusion of patients with non-neurological cause of death.**

|  | Prediction of poor outcome | | Prediction of good outcome | | AUC |
| --- | --- | --- | --- | --- | --- |
| Model parameters | **Sensitivity** | **Specificity** | **Sensitivity** | **Specificity** |  |
| Full dataset (N = 100) |  |  |  |  |  |
| EEG + SSEP | 25% (13-38) | 100% (100-100) | 36% (21-50) | 94% (85-100) | 0.728 (0.649-0.807) |
| EEG + SSEP + ONSD | 41% (22-59) | 100% (100-100) | 28% (12-44) | 93% (81-100) | 0.727 (0.589-0.866) |
| Subset (N = 88) |  |  |  |  |  |
| EEG + SSEP | 27% (16-41) | 100% (100-100) | 42% (25-58) | 93% (84-100) | 0.754 (0.671-0.837) |
| EEG + SSEP + ONSD | 50% (29-71) | 100% (100-100) | 32% (14-50) | 92% (79-100) | 0.816 (0.689-0.944) |

*Data are presented as percentages with 95% confidence intervals.*

**Table S3. Likelihood ratios of EEG + SSEP and ONSD measurements based on logistic regression model (EEG + SSEP) and mixed model with random intercept (EEG + SSEP + ONSD) for the full dataset and subset after exclusion of patients with non-neurological cause of death.**

|  | Prediction of poor outcome | | Prediction of good outcome | |
| --- | --- | --- | --- | --- |
| Model parameters | **LR+** | **LR-** | **LR+** | **LR-** |
| Full dataset (N = 100) |  |  |  |  |
| EEG + SSEP | ∞ | 0.75 | 6.00 | 0.68 |
| EEG + SSEP + ONSD | ∞ | 0.59 | 4.00 | 0.77 |
| Subset (N = 88) |  |  |  |  |
| EEG + SSEP | ∞ | 0.73 | 6.00 | 0.62 |
| EEG + SSEP + ONSD | ∞ | 0.50 | 4.00 | 0.74 |

*LR+: positive likelihood ratio, LR-: negative likelihood ratio.*

**Table S4. Results of logistic regression model for prediction of neurological outcome based on EEG and SSEP for the full dataset.**

| Predictor | Estimated | Odds ratio | 95% CI for odds ratio | | P-value |
| --- | --- | --- | --- | --- | --- |
|  | **Coefficient** |  | **Lower** | **Upper** |  |
| Intercept | 0.15 | 1.17 | 0.85 | 1.60 | 0.34 |
| Suppressed EEG | 17.41 | 3.65e+07 | 2.19e-60 | NA | 0.99 |
| Continuous EEG | -1.54 | 0.21 | 7.60e-02 | 0.52 | <0.01* |
| Absent SSEP | 17.41 | 3.65e+07 | 1.16e-17 | NA | 0.99 |

*EEG: electroencephalogram, SSEP: somatosensory evoked potentials, CI: confidence interval. Significant results are indicated by a *.*

**Table S5. Results of mixed effects model for prediction of neurological outcome based on EEG, SSEP, and ONSD measurements for the full dataset.**

| Predictor | Estimated | Odds ratio | 95% CI for odds ratio | | P-value |
| --- | --- | --- | --- | --- | --- |
|  | **Coefficient** |  | **Lower** | **Upper** |  |
| Intercept | 0.12 | 1.13 | 0.55 | 2.33 | 0.73 |
| Suppressed EEG | 17.22 | 3.02e+07 | 0 | Inf | 0.98 |
| Continuous EEG | -1.46 | 0.23 | 0.05 | 1.08 | 0.06 |
| Absent SSEP | 17.43 | 3.73e+07 | 0 | Inf | 0.99 |
| ONSD | 0.21 | 1.23 | 0.75 | 2.03 | 0.41 |
| Day 2 | 0.04 | 1.04 | 0.38 | 2.87 | 0.93 |
| Day 3 | 0.29 | 1.34 | 0.41 | 4.32 | 0.63 |

*EEG: electroencephalogram, ONSD: optic nerve sheath diameter, SSEP: somatosensory evoked potentials, CI: confidence interval.*

**Table S6. Results of logistic regression model for prediction of neurological outcome based on EEG and SSEP for the subset (after exclusion of patients with a non-neurological cause of death).**

| Predictor | Estimated | Odds ratio | 95% CI for odds ratio | | P-value |
| --- | --- | --- | --- | --- | --- |
|  | **Coefficient** |  | **Lower** | **Upper** |  |
| Intercept | -0.30 | 7.44 | 0.53 | 1.04 | 0.09 |
| Suppressed EEG | 18.86 | 1.56e+08 | 2.38e-103 | NA | 0.99 |
| Continuous EEG | -18.27 | 1.16e-08 | NA | 1.85e+23 | 0.99 |
| Absent SSEP | 18.86 | 1.56e+08 | 6.46e-33 | NA | 0.99 |

*EEG: electroencephalogram, SSEP: somatosensory evoked potentials, CI: confidence interval.*

**Table S7. Results of mixed effects model for prediction of neurological outcome based on EEG, SSEP, and ONSD measurements for the subset (after exclusion of patients with a non-neurological cause of death).**

| Predictor | Estimated | Odds ratio | 95% CI for odds ratio | | P-value |
| --- | --- | --- | --- | --- | --- |
|  | **Coefficient** |  | **Lower** | **Upper** |  |
| Intercept | -0.28 | 0.75 | 0.35 | 1.63 | 0.47 |
| Suppressed EEG | 18.68 | 1.39e+08 | 0 | Inf | 1.00 |
| Continuous EEG | -18.20 | 1.25e-08 | 0 | Inf | 1.00 |
| Absent SSEP | 18.94 | 1.69e+08 | 0 | Inf | 1.00 |
| ONSD | 0.28 | 1.32 | 0.74 | 2.34 | 0.35 |
| Day 2 | -0.07 | 0.93 | 0.31 | 2.82 | 0.90 |
| Day 3 | 0.32 | 1.37 | 0.41 | 4.61 | 0.61 |

*EEG: electroencephalogram, ONSD: optic nerve sheath diameter, SSEP: somatosensory evoked potentials, CI: confidence interval.*
